# Supplementary material for: Combinatorial encoding of odors in the mosquito antennal lobe
Source: Nat Commun. 2023 Jun 15;14:3539. doi: 10.1038/s41467-023-39303-w (PMC10272161; doi:10.1038/s41467-023-39303-w)
Supplement: Supplementary file 1 — Supplementary Information [file 41467_2023_39303_MOESM1_ESM.pdf]

Supplementary Information

Combinatorial encoding of odors in the mosquito antennal lobe

Singh et al.

Supplementary Figure 1

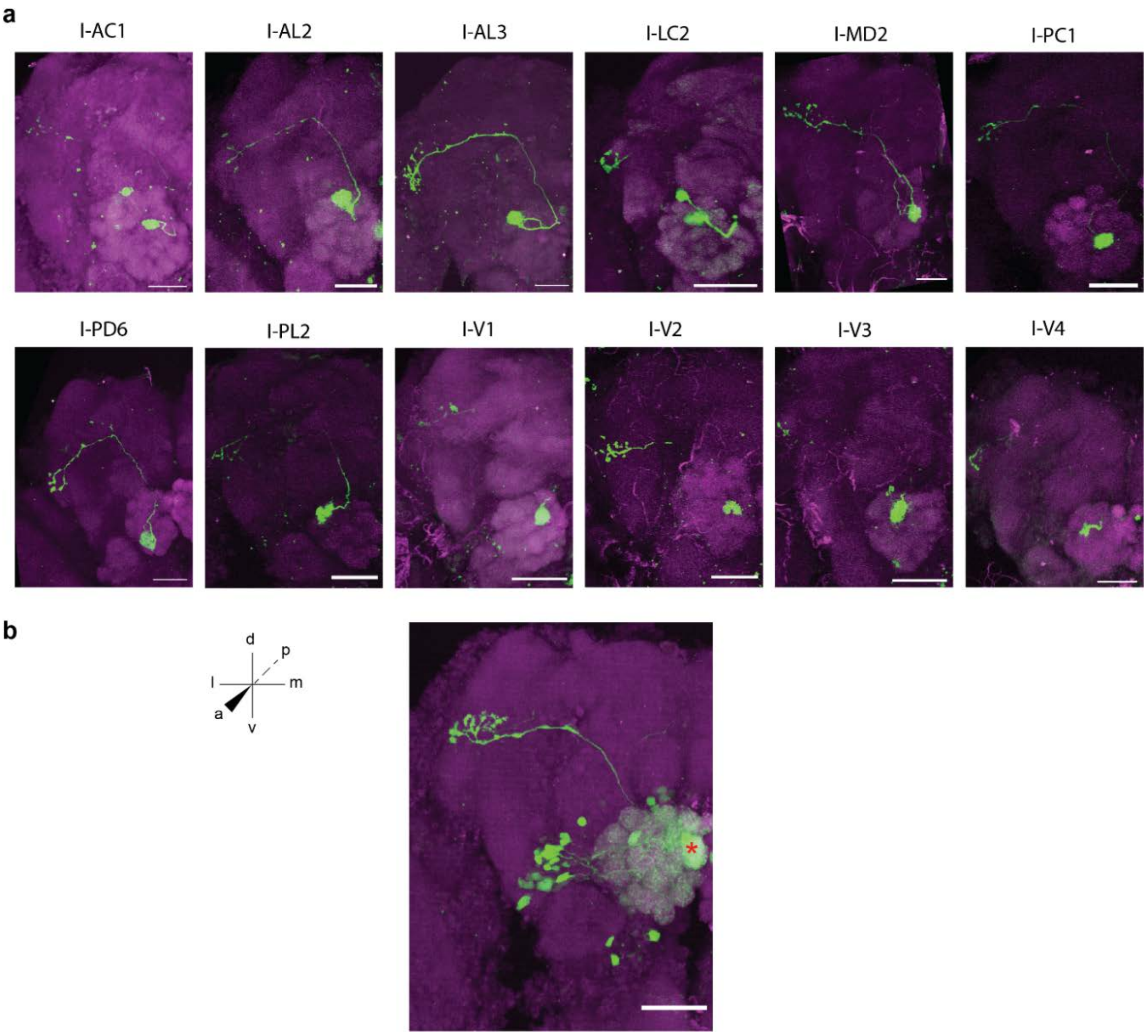

Supplementary Figure 1: Morphology of uniglomerular PN and untargeted labelling of cell bodies

a Examples of uniglomerular PNs (out of 201 total). For each PN, the image shows the morphology in a maximum-intensity projection of the image stack (the region displayed includes ipsilateral AL, mushroom body, and protocerebrum). Some branches are not clearly visible in these projections. Green: biocytin or lucifer yellow used to fill the cell; magenta: Dncad (neuropil marker). **b** The maximum intensity projection from an image stack of one half of an adult female *Aedes aegypti* brain showing the antennal lobe and higher brain regions. One PN was recorded and filled on this side of the brain (corresponding to the brightly filled glomerulus indicated by asterisk and the axonal projection to the higher brain areas). Faint signals in the entire antennal lobe and in some cell bodies in the lateral and ventral clusters are also observed, suggesting the presence of gap junctions between the recorded PN and other LNs/PNs (this phenomenon was observed in 20 cases). Co-ordinate axes: dorsal-ventral (d-v), anterior-posterior (a-p), and medial-lateral (m-l). Green: biocytin; magenta: Dncad (neuropil marker). Scale bars, 50 µm.

Supplementary Figure 2

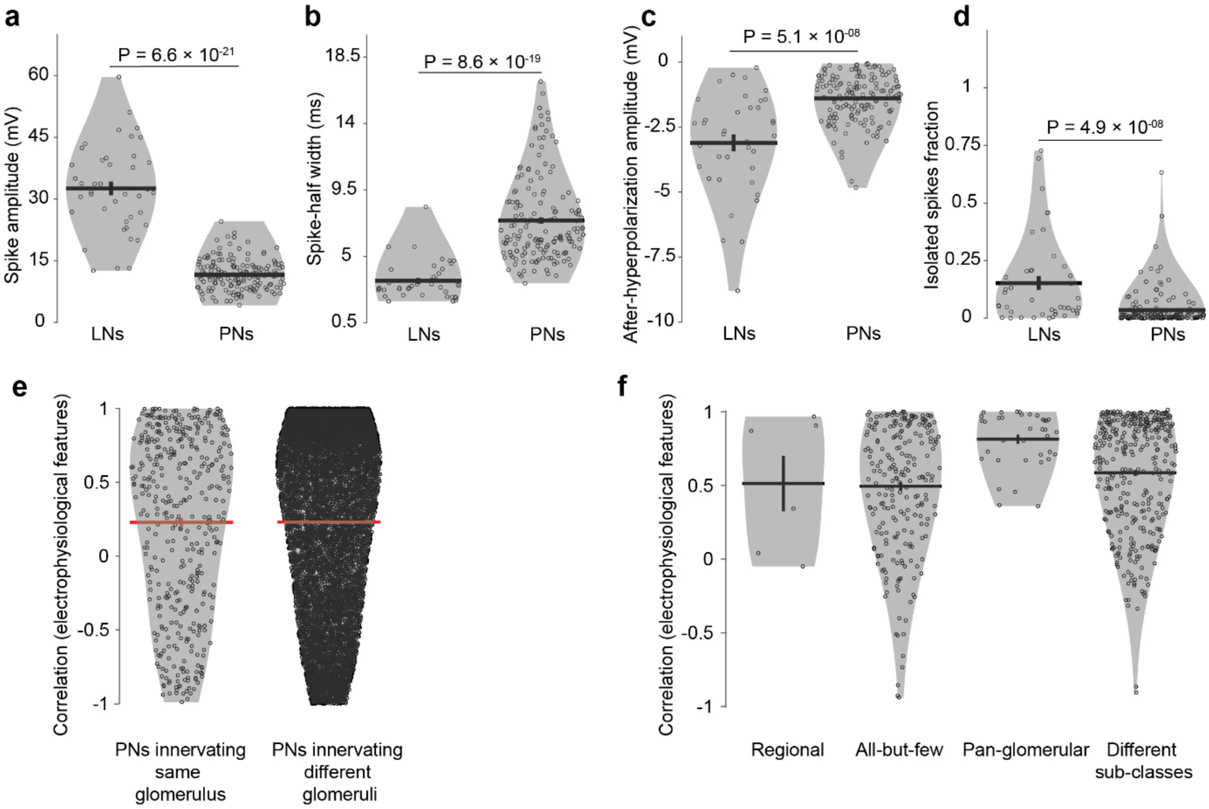

Supplementary Figure 2: Electrophysiological characterization of PNs and LNs

a-d Similar to **Figure 3c-f**, but while **Figure 3c-f** included all isolated spikes present in the recordings, the figures shown here were made with a smaller set of spikes, excluding those spikes that occurred during the odor response duration (2 s from the onset of the odor)**.** LNs (*n* = 42) and PNs (*n* = 170) differed significantly in their values for the 4 electrophysiological features. P-values from two-tailed rank-sum tests are displayed. **e** Correlations between the electrophysiological features of pairs of PNs innervating the same glomerulus (*n* = 418) or innervating different glomeruli (*n* = 10313). **f** Correlations between the electrophysiological features of pairs of LNs within *regional* (*n* = 6), *all-but-few* (*n* = 231), or *pan-glomerular* (*n* = 36) sub-classes, or pairs of LNs belonging to different sub-classes (*n* = 357). Error bar represents s.e.m. Source data are provided as a Source Data file.

Supplementary Figure 3

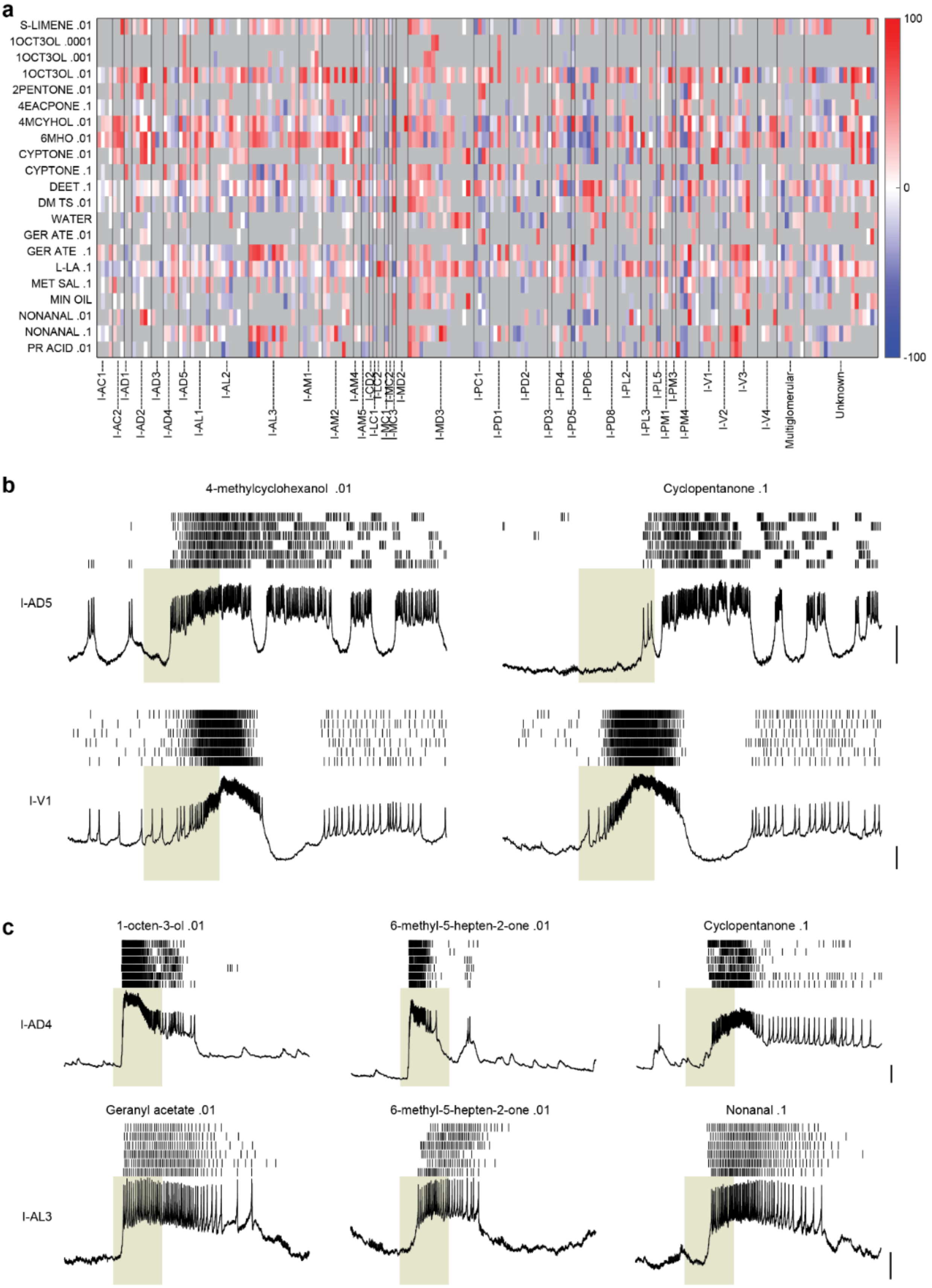

Supplementary Figure 3: Odor responses of projection neurons (PNs)

a This figure is similar to **Figure 4b** but additionally includes multiglomerular PNs, PNs with unknown glomerular identity, and also PNs in which less than 5 odors were tested (such PNs were excluded in **Figure 4b**). The figure includes 201 PNs; only 7 out of 208 PNs that did not show any spikes are excluded. Source data are provided as a Source Data file. **b** Example of PNs showing a delayed onset of odor responses. Individual panels show 5s-long recording snippets (including the trace from the first trial and spike raster for all trials) obtained from two PNs tested with two odors. The onset of spiking in I-AD5 PN in response to cyclopentanone .1 is delayed by ~800 ms after the start of the odor delivery while the response of the same PN to 4-methylcyclohexanol .01 starts much earlier. In contrast, in the I-V1 PN, the response to cyclopentanone .1 starts earlier than the response to 4-methylcyclohexanol .01**.** **c** More examples of PNs showing odor-specific delays in responses**.** Scale bars, 10 mV.

Supplementary Figure 4

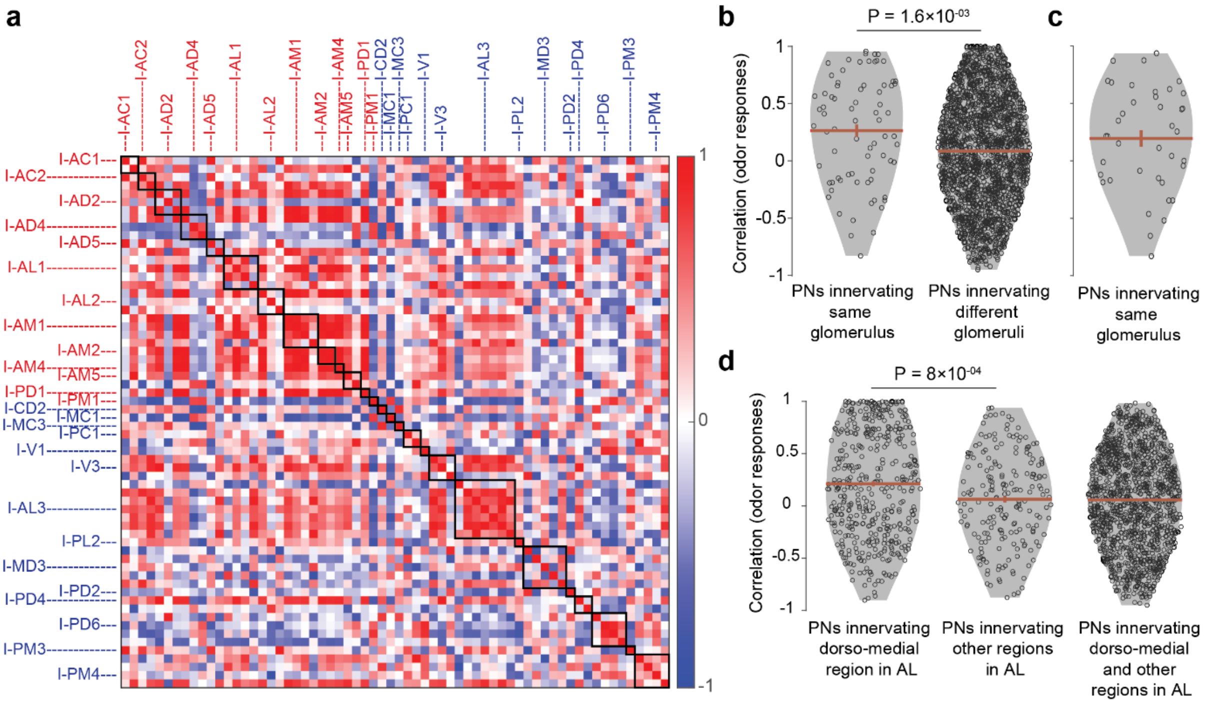

Supplementary Figure 4: Odor response similarity between PNs

a Pairwise correlations between vectors of odor responses of PNs. Responses of 64 PNs to 6 frequently tested odors were used. PNs are arranged according to glomerular identity; boxes along the diagonal indicate PNs belonging to the same glomerulus; dorsomedial glomeruli shown in red font and other glomeruli shown in blue font. **b** Comparison of the correlation values from **(a)** for PN pairs innervating the same glomerulus (left, n = 74) or innervating different glomeruli (right, n = 1942). **c** Similar to **(b)** but using only those PNs whose glomerular identity confidence score (see **Methods**) was ≥ 3.5. Applying this threshold reduced the number of PNs from 64 to 51 and the number of homotypic PN pairs from 74 to n=39 (i.e., approximately half). **d** Comparison of the correlation values from **(a)** for PN pairs with both innervating the dorsomedial glomeruli (left, n = 435); both innervating other glomeruli (middle, n = 200); and one PN innervating a dorsomedial glomerulus and one PN innervating a non-dorsomedial glomerulus (n = 1381) within AL. Red lines: means, error bars: s.e.m. P-values from rank-sum tests are displayed. Source data are provided as a Source Data file.

Supplementary Figure 5

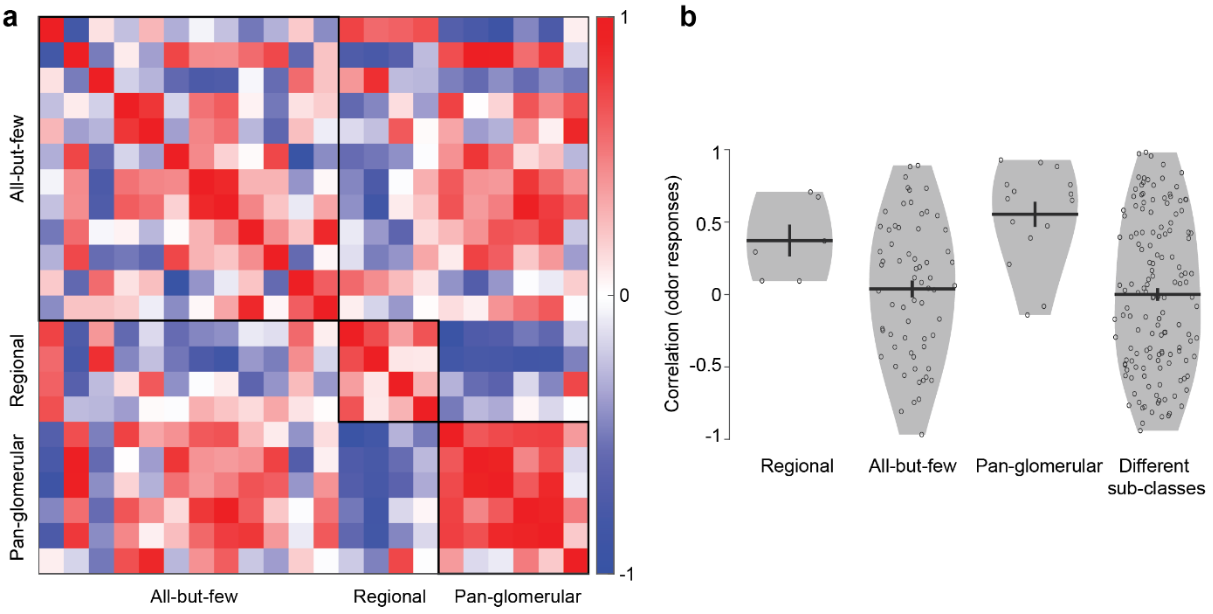

Supplementary Figure 5: Odor response similarity between morphological sub-classes of LNs

a Pairwise correlations between vectors of odor responses of LNs. Responses of 22 LNs to 6 frequently tested odors were used. Boxes indicate morphological sub-classes of LNs. **b** Comparison of the correlation values from **(a)** for pairs of LNs within *regional* (n = 6), *all-but-few* (n = 66), or *pan-glomerular* (n = 15) sub-classes, or pairs of LNs belonging to different sub-classes (n = 144). Black lines: means, error bars: s.e.m. Source data are provided as a Source Data file.

Supplementary Figure 6

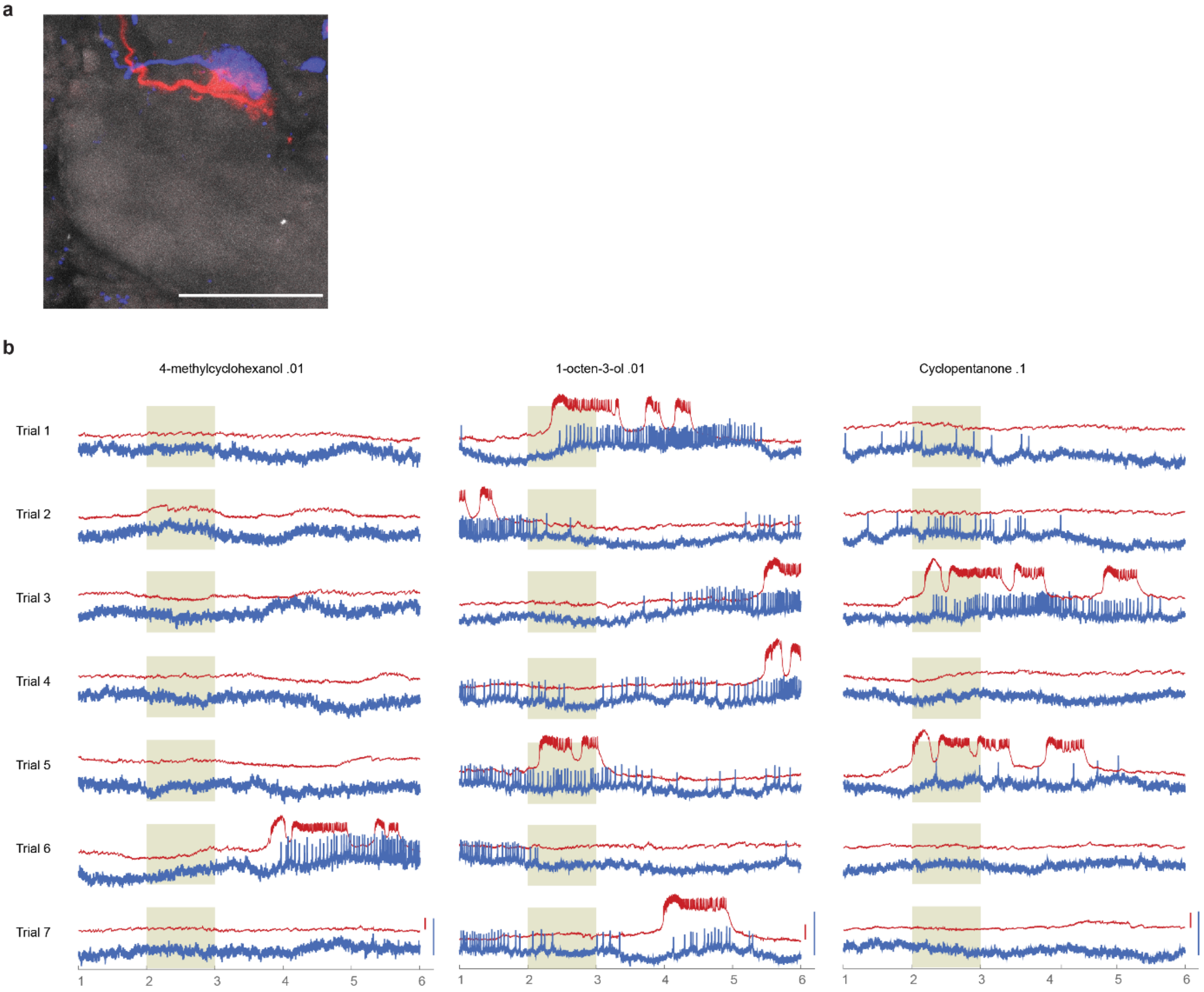

Supplementary Figure 6: Simultaneous recordings from I-AL3 and I-PL2 PNs

a Maximum intensity projection of the image stack showing the glomerular innervations of the two PNs recorded simultaneously in one experiment. Note that the two glomeruli are adjacent to each other (they appear overlapping in the flattened stack but are non-overlapping in 3-D). Blue: I-AL3, red: I-PL2, grey: Dncad (neuropil marker). Scale bar, 50 µm. **b** Simultaneous recordings from an I-AL3 (blue) and I-PL2 (red) PN in response to odors known to activate palp ORNs. Grey shaded region indicates the 1-s odor stimulation period. Scale bars, blue: 5 mV; red: 10 mV

Supplementary Figure 7

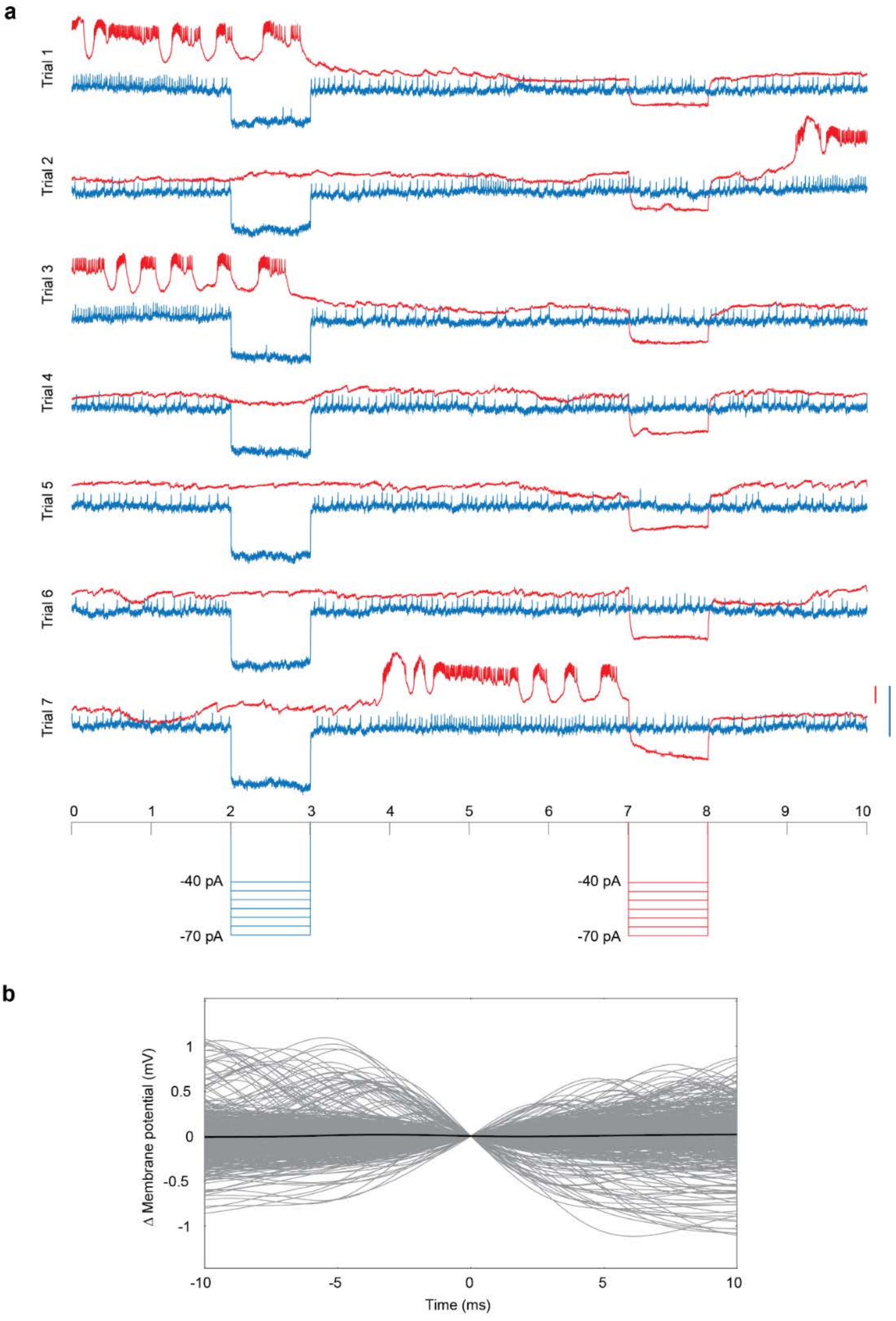

Supplementary Figure 7: Indirect lateral interaction between I-AL3 and I-PL2 PNs

a Simultaneous recording from an I-AL3 (blue) and an I-PL2 (red) PN. Pulses of hyperpolarizing current (-40 pA in the first trial, increasing in steps of -5 pA in every trial) were injected during 2-3 s in I-AL3 (blue) and during 7-8 s in I-PL2 (red). No effect is seen in either cell when the other was hyperpolarized. Scale bars, blue: 5 mV; red: 10 mV **b** Spike-triggered average of the I-AL3 PN's membrane potential, triggered on the I-PL2 PN spikes, did not provide any evidence of a direct connection from the I-PL2 PN to the I-AL3 PN. Grey: individual traces; black: average of all traces. Source data are provided as a Source Data file.

Supplementary Figure 8

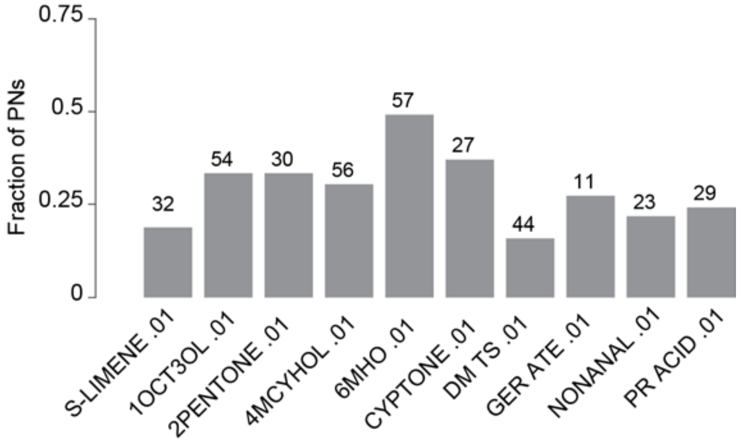

Supplementary Figure 8: Fraction of PNs activated by odors more strongly than mineral oil

This figure is similar to **Figure 7a**, but uses a different statistical criterion to classify a PN-odor pair as 'responding' and 'non-responding'. This analysis is performed for odors diluted in mineral oil and compares the odor response with the mineral oil response to make the classification. We divided the response duration into two 1-s bins and calculated the change in spiking rate in each bin by subtracting the background spiking rate from the number of spikes in the bin. We compared the change in the spiking rate in each bin over all trials for the odor and for mineral oil in the same cell using a two-sided signed-rank test. If the test showed a significantly high or low firing rate in either bin compared to the mineral oil at the p-value threshold of 0.05, the cell-odor pair was labeled as 'responding'. The number above each bar indicates the number of cells tested with the odor. Cells in which the mineral oil response was not tested are excluded. Source data are provided as a Source Data file.

Supplementary Figure 9

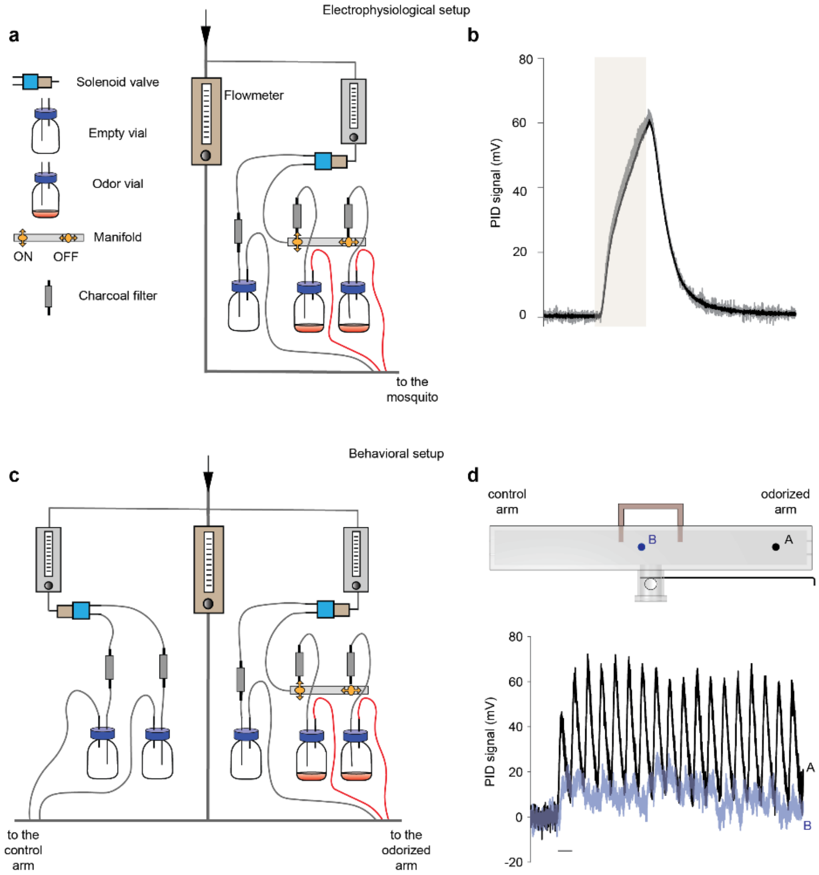

Supplementary Figure 9: Odor delivery setup for electrophysiological and behavioral experiments

a Schematic showing the odor delivery setup used for electrophysiological experiments. A stream of compressed air (2L/min) is divided into background stream (1.8L/min, 90%) and a flexible stream (0.2L/min, 10%) using flowmeters. A solenoid valve switches the flexible stream between an empty vial and the odor vials. Odor vials are connected to a manifold that allowed the flexible stream to pass through one vial at a time. The desired odor is delivered by rotating the stopcock on the manifold. A charcoal filter placed at the inlet of the vials prevents contamination. **b** PID signal for an odor (6-methyl-5-hepten-2-one .01) in the electrophysiology rig when the PID probe was kept at the same position where the mosquito would normally be kept. Grey traces, different trials; black trace, average of all trials. Grey shaded region indicates the 1-s odor stimulation period. **c** Schematic showing the odor delivery setup used for behavioral experiments. The odorized size is similar to the electrophysiology setup. An air stream of 5L/min is divided into background stream (4.5L/min, 90%) and a flexible stream (0.5L/min, 10%) using flowmeters (higher flow rates were used to compensate for differences in the physical dimensions of the behavioral and the electrophysiological setup). The control side is also the same except that an empty vial is used in place of the odor vials. **d** PID signal recorded for the same odor (6-methyl-5-hepten-2-one .01) when a small tube connected to the PID was placed inside the behavioral chamber (top). The black trace corresponds to PID signal recorded when the tube was placed inside the odorized arm (position A). Note that the PID amplitude in electrophysiological set-up and in the odorized arm is similar (approx. 60 mV). The blue trace corresponds to PID signal recorded when the tube was placed slightly towards the control side from the center of the chamber but facing the odorized arm (position B). In behavioral experiments, odor was delivered in pulses of 1s ON:4s OFF. Scale bar represents 5 s. Source data are for **b,d** provided as a Source Data file.

Supplementary Figure 10

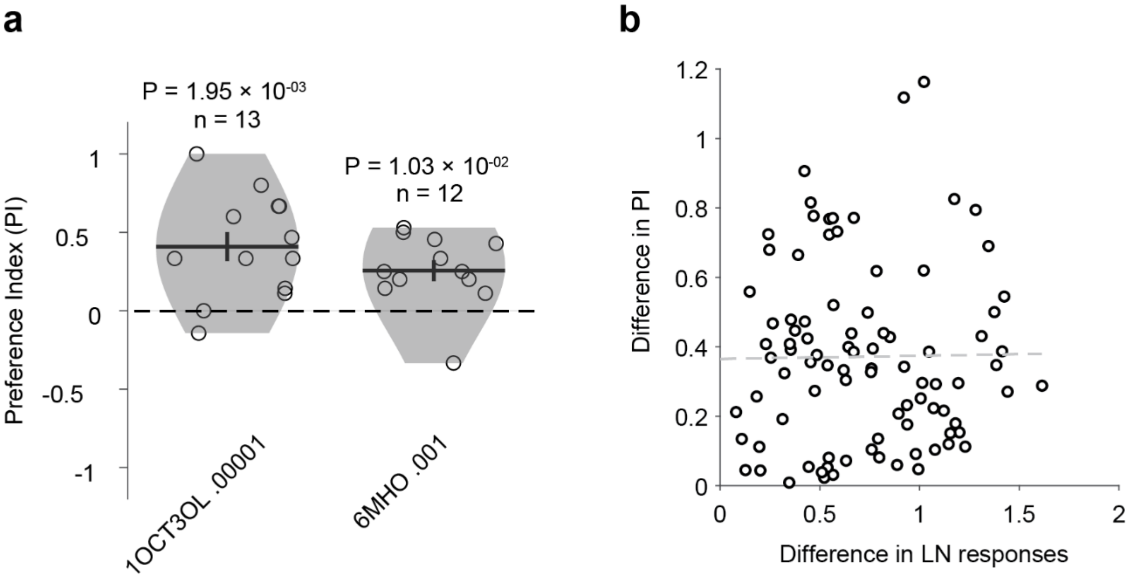

Supplementary Figure 10: Behavioral responses to odors

a Preference index (PI) values observed for 1-octen-3-ol 0.00001 and 6-methyl-5-hepten-2-one 0.001 (lower concentrations than reported in **Figure 8** in the main text). P-values are calculated from two-sided signed-rank tests comparing PI to 0; n (number of repeated experiments) is indicated above each plot. Black lines: means, error bars: s.e.m. **b** This figure is similar to **Figure 8c** but is made for LNs instead of PNs. Across odor pairs, the difference in preference indices (PI) is not correlated with the difference in LN responses (R = 0.013, P = 0.9, n = 90 odor pairs). Source data are provided as a Source Data file.

Supplementary Table 1: Odorants used in the study

Odorant	Acronym	Chemical group	Ecological significance
1-octen-3-ol	1OCT3OL	alcohol	component of human odor
4-methylcyclohexanol	4MCYHOL	alcohol	oviposition attractant
Nonanal	NONANAL	aldehyde	component of human odor
4’ethylacetophenone	4EACPONE	alkyl-phenylketones	aggregation pheromone
L-lactic acid	L-LA	alpha-hydroxy acid	component of human odor
Propionic acid	PR ACID	carboxylic acid	component of human odor
Methyl salicylate	MET SAL	benzoate ester	plant-derived
2-pentanone	2PENTONE	ketone	component of human odor
6-methyl-5-hepten-2-one	6MHO	ketone	component of human odor
Cyclopentanone	CYPTONE	ketone	mimicking carbon dioxide
(S)-limonene	S-LIMENE	monoterpene	plant-derived
Geranyl acetate	GER ATE	monoterpenoid	plant-derived
N,N-diethyl-meta-toluamide	DEET	N,N-dialkylamide	synthetic repellent
Dimethyl trisulfide	DM TS	sulfide	component of human odor
