# Supplementary material for: Combinatorial encoding of odors in the mosquito antennal lobe
Source: Nat Commun. 2023 Jun 15;14:3539. doi: 10.1038/s41467-023-39303-w (PMC10272161; doi:10.1038/s41467-023-39303-w)
Supplement: Supplementary file 3 — Reporting Summary [file 41467_2023_39303_MOESM3_ESM.pdf]

nature portfolio

Corresponding author(s): Nitin Gupta

Last updated by author(s): May 25, 2023

Reporting Summary

Nature Portfolio wishes to improve the reproducibility of the work that we publish. This form provides structure for consistency and transparency in reporting. For further information on Nature Portfolio policies, see our Editorial Policies and the Editorial Policy Checklist.

Statistics

For all statistical analyses, confirm that the following items are present in the figure legend, table legend, main text, or Methods section.

n/a	Confirmed	
☐	☒	The exact sample size (*n*) for each experimental group/condition, given as a discrete number and unit of measurement
☐	☒	A statement on whether measurements were taken from distinct samples or whether the same sample was measured repeatedly
☐	☒	The statistical test(s) used AND whether they are one- or two-sided<br>*Only common tests should be described solely by name; describe more complex techniques in the Methods section.*
☒	☐	A description of all covariates tested
☒	☐	A description of any assumptions or corrections, such as tests of normality and adjustment for multiple comparisons
☐	☒	A full description of the statistical parameters including central tendency (e.g. means) or other basic estimates (e.g. regression coefficient) AND variation (e.g. standard deviation) or associated estimates of uncertainty (e.g. confidence intervals)
☐	☒	For null hypothesis testing, the test statistic (e.g. *F*, *t*, *r*) with confidence intervals, effect sizes, degrees of freedom and *P* value noted<br>*Give P values as exact values whenever suitable.*
☒	☐	For Bayesian analysis, information on the choice of priors and Markov chain Monte Carlo settings
☒	☐	For hierarchical and complex designs, identification of the appropriate level for tests and full reporting of outcomes
☐	☒	Estimates of effect sizes (e.g. Cohen's *d*, Pearson's *r*), indicating how they were calculated

Our web collection on statistics for biologists contains articles on many of the points above.

Software and code

Policy information about availability of computer code

Data collection	Electrophysiological Data: Pclamp10<br>Histological Data: NIS-C version 5.1
Data analysis	Electrophysiological and behavioral data: Matlab 2020b<br>Histological Data: ImageJ version 1.53, Neutube 1.0, and R<br>The classification analysis was performed using a custom library available at https://github.com/neuralsystems/temporal_classification. Code used for the classification of LNs and PNs based on electrophysiology features and the code used for generating trajectories of PN population responses are available at https://github.com/neuralsystems/NatComm2023 (https://doi.org/10.5281/zenodo.7969005)

For manuscripts utilizing custom algorithms or software that are central to the research but not yet described in published literature, software must be made available to editors and reviewers. We strongly encourage code deposition in a community repository (e.g. GitHub). See the Nature Portfolio guidelines for submitting code & software for further information.

Data

Policy information about availability of data

All manuscripts must include a data availability statement. This statement should provide the following information, where applicable:

- Accession codes, unique identifiers, or web links for publicly available datasets
- A description of any restrictions on data availability
- For clinical datasets or third party data, please ensure that the statement adheres to our policy

The morphological reconstructions of PNs registered to the template brain are available at https://github.com/neuralsystems/MosquitoAL. Source data are provided with this paper.

Research involving human participants, their data, or biological material

Policy information about studies with human participants or human data. See also policy information about sex, gender (identity/presentation), and sexual orientation and race, ethnicity and racism.

Reporting on sex and gender	N/A
Reporting on race, ethnicity, or other socially relevant groupings	N/A
Population characteristics	N/A
Recruitment	N/A
Ethics oversight	N/A

Note that full information on the approval of the study protocol must also be provided in the manuscript.

Field-specific reporting

Please select the one below that is the best fit for your research. If you are not sure, read the appropriate sections before making your selection.

☒ Life sciences ☐ Behavioural & social sciences ☐ Ecological, evolutionary & environmental sciences

For a reference copy of the document with all sections, see nature.com/documents/nr-reporting-summary-flat.pdf

Life sciences study design

All studies must disclose on these points even when the disclosure is negative.

Sample size	No sample size calculation was performed before experiments. The sample sizes were chosen according to the accepted practices in the field.
Data exclusions	Cells showing no spikes in background and in response to stimuli (current or odor) were excluded. Low quality recordings were excluded for elctrophysiological classification analysis.
Replication	For electrophysiological experiments, we tested each odor for at least 6 trials within a neuron and if the number was less than 6, then it was excluded from analysis. For behavioral experiments, at least 12 experiments were performed for each odor. Experiments in which the number of responding mosquitoes was less than 5 were excluded.
Randomization	For electrophysiological experiments, sequence of odor stimuli was randomized. For behavioral experiments, the side of the odorized was randomized. Animals were randomly chosen for behavioral experiments, and each animal was used for behavioral experiment with only one odor.
Blinding	Blinding was not applicable to the behavioral experiments performed with wild-type mosquitoes in this study.

Reporting for specific materials, systems and methods

We require information from authors about some types of materials, experimental systems and methods used in many studies. Here, indicate whether each material, system or method listed is relevant to your study. If you are not sure if a list item applies to your research, read the appropriate section before selecting a response.

Materials & experimental systems

n/a	Involved in the study	
☐	☒	Antibodies
☒	☐	Eukaryotic cell lines
☒	☐	Palaeontology and archaeology
☐	☒	Animals and other organisms
☒	☐	Clinical data
☒	☐	Dual use research of concern
☒	☐	Plants

Methods

n/a	Involved in the study	
☒	☐	ChIP-seq
☒	☐	Flow cytometry
☒	☐	MRI-based neuroimaging

Antibodies

Antibodies used

Primary antibodies: rat anti-DN-cadherin (1:30 dilution, DN-EX #8, Developmental Studies Hybridoma Bank), rabbit anti-GABA (1:500 dilution, A2052, Sigma-Aldrich) and rabbit anti-Lucifer yellow (1:200 dilution, A5750, Molecular Probes).
Secondary antibodies: goat anti-rat Alexa 405 (1:500 dilution, ab175671, Abeam), goat anti-rabbit Alexa 633 (1:500 dilution, A21070, Molecular Probes), goat anti-rabbit Alexa 488 (1:500 dilution, A11008, Molecular Probes), Streptavidin Alexa 488 (1:1QA5 dilution, 511223, Molecular Probes) or Streptavidin Alexa 568 (1:10A5 dilution, 511226, Molecular Probes)

Validation

These are commercially available antibodies that have been widely used previously in insects.

Animals and other research organisms

Policy information about studies involving animals; ARRIVE guidelines recommended for reporting animal research, and Sex and Gender in Research

Laboratory animals

Mosquitoes (Aedes aegypti (Linnaeus) Liverpool strain, 4-8 days old non-blood fed females) for experiments and mice (Mus musculus BALB/c) for blood feeding. Mice were housed in the same environmental conditions as mosquitoes.

Wild animals

No wild animals were used.

Reporting on sex

Only female mosquitoes were used in this study. Female mosquitoes are larger in size with less bushier antennae. These features were used for segregation. Mice were only used for breeding of mosquitoes as a source of blood, but not used for any specific experiments, therefore the gender for the mice is not relevant.

Field-collected samples

No field-collected samples were used.

Ethics oversight

Mice handling was done as per the approved protocol of the Institute Animal Ethics Committee of the Indian Institute of Technology Kanpur.

Note that full information on the approval of the study protocol must also be provided in the manuscript.
